# Detection the severity of organophosphate intoxication using sensitive serum biomarkers S100B and amyloid β (Aβ) in Egyptian subjects

**DOI:** 10.1007/s11356-023-29748-3

**Published:** 2023-09-15

**Authors:** Mostafa M. Elhady, Sara A. Adly, Husam A. Elshebiny, Said S. Moselhy

**Affiliations:** 1 Biochemistry Department, Faculty of Science, Ain Shams University, Cairo, Egypt; 2 Poison Control Center, Faculty of Medicine, Ain Shams University, Cairo, Egypt

**Keywords:** Organophosphate, Severity, S100B, Amyloid β, Intoxication

## Abstract

Organophosphate (OP) is a compound considered the main leading cause of morbidity and mortality from poisoning worldwide. Serum pseudocholinesterase was evaluated as a diagnostic indicator; it cannot be used to monitor therapy or severity of the intoxication. The rationale of the current study was to evaluate sensitivity, specificity, and cut-off values of serum S100B and amyloid β for neurological affection severity. This study was carried out on sixty OP-impaired patients; in addition, 20 normal controls were included. Serum liver and kidney function tests, malondialdehyde, pseudocholinesterase, and the levels of S100B and amyloid β (Aβ) were determined. Data showed that Pearson’s analysis indicated that the serum level of S100B was positively correlated with Aβ. On the contrary, the activity of pseudocholinesterase was negatively correlated with both of S100B and Aβ. Serum ALT, AST, creatinine, urea, acetylcholine, and MDA levels were elevated while pseudocholinesterase activity was reduced in moderate and severe OP intoxication versus control. A drastic elevation (*p*<0.001) in the levels of S100B and Aβ was performed in the patient group suffering from OP intoxication versus the normal group. The diagnostic statistical validation of targeted parameters in distinguishing between moderate OP intoxication and control clarifies that S100B displayed the best AUC (0.997) followed by Aβ (AUC=0.992), while the diagnostic veracity of S100B and Aβ in setting apart severe OP-intoxicated and normal subjects stated the symmetric efficacy of potential markers. It was concluded that the significant changes in the levels of S100B and Aβ were directly proportional to the degree of severity of OP intoxication.

## Introduction

Suicide by pesticides is considered complicated public problems, particularly in developed countries (Jacob 2017). Due to low price and availability of this pesticide poison, it increased the rate of morbidity and mortality if not rapidly managed (Eddleston et al. 2008). In addition, with delayed diagnosis and intervention, consequences of complications increased (Manoranjitham et al. 2010). Organophosphate (OP) poisoning remains a frequent cause for hospitalization in developing countries (Senarathna et al. 2012). The traditional approaches to clinical symptoms in acute OP poisoning has focused on receptor-specific effects on muscarinic, nicotinic, and central nervous system (CNS) receptors resulting in diverse features and outcomes (Noshad 2007). This conventional classification of clinical features is useful given that muscarinic effects are reversed by atropine while nicotinic neuromuscular effects are not affected (Anand et al. 2009).

The OP toxication symptoms include urination, salivary excretion, lacrimation, and emesis. In some cases, delayed symptoms appear either after an initial period of intense cholinergic effect. However, acute symptoms including neuronal effects (e.g., neuromuscular weakness) may have delayed development (karki et al. 2004). OPs can be fatal within minutes of exposure. Mortality depends upon the amount taken, route of ingestion, general health reputation, and prompt diagnosis. The first aid in treatment of OP intoxication is atropine with fluid intravenous administration and oxygen supply therapy (Eddleston et al. 2008). For rapid management of OP side effects, a sensitive biomarker that would allow easier identification of patients with OP intoxication is needed. The levels of serum β-glucuronidase and paraoxonase were measured both diagnosis and monitoring. However, these markers are very expensive (Ceja et al. 2020). ACE is found in red cells and neurons while pseudocholinesterase is present in the serum, plasma, liver, pancreas, heart, and other tissues. Previous studies have shown that serum pseudocholinesterase can be used as a diagnostic marker for OP intoxication with advantage over the evaluation of its activity in erythrocytes in that it is simpler and more accurate to measure (Tareg 2001). In OP acute intoxication, serum cholinesterase was inhibited to half activity. The OP severity depends on serum cholinesterase inhibition rate. But its role as a gold standard for OP diagnosis and treatment monitoring is minimal, so cheap and easily measurable biomarkers having diagnostic value are urgently needed. It would also be beneficial to correlate the levels of these biomarkers with the severity of the disease and its applicability to tracking responses to remedy. The protein S100B belongs to a family of acidic proteins that bind calcium ions in many tissues in the body. Normally, serum S100B protein does not exceed 100 ng/L; it was elevated during neural damage (Chen et al. 2022). Amyloid β (Aβ) accumulation leads to clinical manifestations ranging from dementia to hemorrhage (Miller-Thomas et al. 2016). This accumulation contributed to neurological disorders, the formation of neurofibrillary tangle, and progressive neuronal loss. Aβ plaques caused inflammation and dystrophic neurites (Braak et al. 2011). The rationale of the current study was to evaluate sensitivity, specificity, and cut-off values of serum S100B and amyloid β for neurological affection severity. In this study, we investigated the validity of serum sensitive biomarker S100B combined with serum Aβ protein in the differentiation the severity of OP intoxication for early intervention and management.

## Subjects and methods

### Study participants

This study was carried out according to ethical committee, Ain Shams University. A consent form was taken from all subjects included in the study. Sixty adult unrelated Egyptian patients admitted to Poison Control Center (PCC), Ain Shams University Hospital, suffering from OP intoxication who met the criteria of OP poisoning diagnosis were enrolled in the current research. In addition, 20 healthy subjects recruited from patient relatives were enrolled as controls. The control participants were included in the study after confirming that they were not suffering from any previous OP intoxication. The exclusion criteria include diabetes, hypertension, thyrotoxicosis, or under any therapeutic agents.

Clinical examination was done systematically where the vital data respiratory rate, heart rate, blood pressure, body temperature, and any systemic abnormalities were monitored. The Peradeniya OP Poisoning (POP) Scale proposed by Senanayake et al. (1993) was relied upon in the categorization of the clinical severity of OP intoxication (Table 1).

**Table 1:** The Peradeniya scores of OP poisoning

	Clinical criteria	Score
Size of pupil	> 2 mm < 2 mm Pin-point	0 1 2
Rate of respiration	< 20/min > 20/min > 20/min with central cyanosis	0 1 2
Rate of heartbeat	> 60/min 41–60/min < 40/min	0 1 2
Prescence or absence of fasciculation	None Present, generalized, or continuous Both, generalized, and continuous	0 1 2
Consciousness level	Conscious and rationale Impaired response to verbal commands No response to verbal commands	0 1 2
Occurrence of seizures	Absent Present	0 1

Mild if score 0 to 3, moderate 4 to 7, and severe 8 to 11

### Biochemical analyses

Five milliliters of venous blood was collected from each participant and centrifuged at 2500 × g for 10 min; sera were separated and kept at − 40 °C till analysis.

The activities of serum ALT and AST were determined kinetically using commercial kits (Bio Diagnostics, Egypt). Serum creatinine level was quantified according to the method described by Henry et al. 1960). Kassirer method was used to assess the concentration of urea level (Kassirer et al. 1971). Acetylcholine and malondialdehyde levels were assessed according to the protocols described in ELISA kits provided by Bioassay Technology Laboratory (cat no. E1371Hu and cat no. E129201Hu). The pseudocholinesterase activity was estimated automatically using Roche/Hitachi cobas C system. ELISA kits for assaying the levels of S100B (cat no. E3887Hu) and Aβ (cat no. E1230Hu) were provided by Bioassay Technology Laboratory.

### Statistical analysis

SPSS version 23.0 was utilized for statistical analysis (IBM Corp, NY, USA). Data was expressed as mean ± standard deviation (SD). One-way analysis of variance (ANOVA) used for comparison. Spearman correlations (*r*) was evaluated. Receiver operating curve was done for cut-off values, validity sensitivity, specificity, and accuracy.

## Results

### Social and demographic data

Social and demographic data presented in Table 2 showed that patient groups included 32 males and 28 females, age of 27.7 ± 12.2 years. With regard to the mode of poisoning, suicidal ingestion was observed in 50 cases (83%) while accidental attempt was evident in 10 cases (17%). Concerning the route of exposure, oral intoxication was noticed in all cases (100%). Concerning POP scoring system, it was between 4 and 7 in 30 cases (50%) that represent moderate OP intoxication sufferers, while POP score was between 8 and 11 in 30 patients (50%) which represent severe cases.

**Table 2:** Social and demographic data

		Control	Total OP	Moderate	Severe
Sex	Male	20 (50%)	32 (53.3%)	17 (56.6%)	15 (50%)
	Female	12 (50%)	28 (46.6%)	13 (43.3%)	15 (50%)
	Mean ± SD	28.3 ± 11.6	27.7 ± 12.2	27.4 ± 12.6	28.1 ± 11.8
Mode of exposure	Accidental		10 (16.6%)	7 (23.3%)	3 (10%)
	Suicidal		50 (83.3%)	23 (76.6%)	27 (90%)
Route of exposure	Oral		60 (100%)	0 (0%)	0 (0%)
	IV		0 (0%)	0 (0%)	0 (0%)
Delay time	Range		0.5–12 h	0.5–4 h	6–12 h
	Mean ± SD		5.6 ± 6.4	1.8 ± 2.2	9.4 ± 3

### General clinical manifestations

Miosis was considered the most common sign in all patients where it was observed in 98.3% of patients; fasciculation was noticed in 66.6% of OP-intoxicated patients; tachypnea was characterized in about 28.3% of the patient group. In the meantime, cyanosis was rated as the least attribute that was remarkable in 16.6% of patients (Table 3).

**Table 3:** Signs and symptoms at presentation

		Total OP	Moderate	Severe
General manifestation	Fasciculation	40 (66.6%)	15 (50%)	24 (80%)
	Cyanosis	10 (16.6%)	0 (0%)	10 (33.3%)
	Miosis	59 (98.3%)	29 (96.6%)	30 (100%)
CNS manifestation	Seizure	11 (18.3%)	0 (0%)	11 (36.6%)
	Agitation	11 (18.3%)	0 (0%)	11 (36.6%)
	Disrupted conscious	34 (56.6%)	4 (13.3%)	30 (100%)
GIT manifestation	Vomiting	25 (41.6%)	10 (33.3%)	15 (50%)
	Nausea	25 (41.6%)	10 (33.3%)	15 (50%)
	Diarrhea	30 (50%)	13 (43.3%)	17 (56.6%)
Respiratory manifestation	Respiratory distress	15 (25%)	0 (0%)	15 (50%)
	Tachypnea	17 (28.3%)	0 (0%)	17 (34%)
	Mechanical ventilation	15 (25%)	0 (0%)	15 (50%)
Clinical outcome	Complete recovery	56 (93.3%)	30 (100%)	26 (86.6%)
	Death	4 (6.7%)	0 (0%)	4 (13.3%)

### Central nervous system and gastrointestinal manifestations

As regards CNS symptoms, the disturbed conscious level was recorded in 34 patients (56.6%), while agitation was noticed in 11 OP sufferers (18.3%). Meanwhile, seizures were observed in 11 intoxicated patients. Pertaining to GIT manifestations, vomiting, nausea, and diarrhea were noticed in 25, 25, and 30 patients respectively.

### Respiratory manifestations and clinical outcomes

Respiratory aspects revealed that respiratory distress was observed in 25% of patients. Fifteen cases of OP-intoxicated patients needed mechanical ventilation. As regards the clinical outcomes, 93.3% of patient of studied cases were fully recovered and three patients (6.7%) died.

### Laboratory findings in OP-intoxicated sufferers on admission

Data presented in Table 4 demonstrated that the activities of ALT and AST were highly significantly increased in moderate and severe cases compared to their levels in control cases.

**Table 4:** Laboratory findings in control and organophosphate-intoxicated subjects on admission

Group Parameter		Control	Total OP	Moderate	Severe	*p* value	
	Range	12–25	13–60	13–34	15–60	0.000ª 0.000^b^ 0.000^c^ 0.000^d^	
ALT (U/l)	Mean ± SD	18.53 ± 3.16	27.38 ± 1.33	24.27 ± 4.68	30.5 ± 8.21		
AST (U/l)	Range	16–25	20–85	20–32	25–85	0.002^a^ 0.002^b^ 0.000^c^ 0.000^d^	
	Mean ± SD	20.27 ± 2.66	30.67 ± 9.29	24.27 ± 4.68	35.33 ± 10.78		
Urea (mg/dL)	Range	15–22	12–43	12–38	18–43	0.008^a^ 0.689^b^ 0.000^c^ 0.008^d^	
	Mean ± SD	19.1 ± 2.09	21.88 ± 5.41	19.53 ± 4.74	24.23 ± 5.07		
Creat. (mg/dL)	Range	0.6–1.2	0.6–1.4	0.6–1.2	0.6–1.4	0.034^a^ 0.732^b^ 0.001^c^ 0.002^d^	
	Mean ± SD	0.83 ± 0.18	0.91 ± 0.20	0.83 ± 0.18	0.99 ± 0.2		
PCE (U/l)	Range	5591–9910	124–2948	1067–2948	124–994	0.000ª 0.000^b^ 0.000^c^ 0.000^d^	
	Mean ± SD	7520.97 ± 191	1056.9 ± 540	1499.23 ± 384	614.57 ± 202		
Acetylcholine (μg/mL)	Range	1.2–3.4	7.2–26.31	7.2–16.3	12–26.31	0.001ª 0.000^b^ 0.000^c^ 0.000^d^	
	Mean ± SD	2.23 ± 0.58	15.15 ± 4.06	12.44 ± 2.63	17.86 ± 3.38		
MDA (nmol/mL)	Range	0.09–9.8	3.1–8.5	3.1–5.8	4.02–8.5	0.731^a^ 0.000^b^ 0.000^c^ 0.768^d^	
	Mean ± SD	4.37 ± 17.70	5.15 ± 1.22	4.31 ± 0.71	6.00 ± 1.02		
S100B (ng/L)	Range	56.8–135.6	125–488.2	125–290.3	256.6–488.2	0.000ª 0.000^b^ 0.000^c^ 0.000^d^	
	Mean ± SD	83.15 ± 17.24	251.64 ± 81.94	187.28 ± 46.24	316.0 ± 54.31		
Aβ (ng/L)	Range	12–36	30.4–80.3	30.4–61.2	36.5–80.3	0.000ª 0.000^b^ 0.000^c^ 0.000^d^	
	Mean ± SD	21.89 ± 5.48	51.06 ± 11.22	45.9 ± 9.54	56.2 ± 10.50		

*ALT *alanine aminotransferase, *AST* aspartate aminotransferase, *MDA *malondialdehyde, *PCE *pseudocholinesterase. *p* value<0.05 was considered significant. ^a^Difference in distribution between organophosphate intoxication group and controls; ^b^difference in distribution between moderate cases and controls; ^c^difference between severe cases and controls; ^d^difference between severe cases and moderate

Creatinine and urea levels showed significant increases between cases in moderate and severe intoxicated cases compared to the normal group (*p*<0.001). Also, there was highly significant increase in acetylcholine level in moderate and severe groups compared to control (*p*<0.001). OP caused a significant elevation in MDA level in both groups versus the normal group; in contrast, statistical reduction was recorded in pseudocholinesterase activity in moderate and severe OP intoxication compared to its standard activity in the control group (Table 4). The data represented in Table 4 showed a drastic elevation (*p*<0.001) in the levels of S100B and amyloid β in the patient group suffering from OP intoxication versus control. The observed changes in the levels of the different estimated parameters were directly proportional to the degree of severity of organophosphate intoxication.

### The diagnostic validity test of S100B and Aβ in the discrimination between control and OP-intoxicated subjects

The diagnostic validity data revealed that S100B exhibited the superior AUC (0.998) followed by amyloid β (AUC=0.996) in differentiating between OP-intoxicated and control subjects. The diagnostic validation of targeted parameters in distinguishing between moderate OP intoxication and control clarifies that S100B displayed the best AUC (0.997) followed by Aβ (AUC=0.992), while the diagnostic veracity of S100B and amyloid β in setting apart severe OP-intoxicated and normal subjects stated the symmetric efficacy of potential markers (Table 5).

**Table 5:** Diagnostic validity test of S100B and Aβ in the discrimination between different groups

		AUC	Cut-off value	sensitivity	specificity	PPV	NPV	Accuracy
C vs T	S100B	0.998	> 121.2	98.33	96.67	98.4	100.0	99.8%
	Aβ	0.996	> 31	100.0	93.33	98.3	96.7	99.7%
C vs M	S100B	0.997	> 121.2	100.0	96.67	96.8	100.0	99.7%
	Aβ	0.992	> 29	100.0	93.33	93.7	100.0	99.2%
C vs S	S100B	1.000	> 135.6	100.0	100.0	100.0	100.0	100%
	Aβ	1.000	> 36	100.0	100.0	100.0	100.0	100%
M vs S	S100B	0.983	> 266.3	96.67	96.67	96.7	96.7	98.3%
	Aβ	0.764	> 50.1	76.67	66.67	69.7	74.1	76.4%

*C vs T* control OP intoxication; *C vs M* control versus moderate; *C vs S* control versus severe; *M vs S* moderate versus severe; *PPV*,* NPV* positive and negative predictive values; *AUC* area under curve

### Correlation between serum S100B, Aβ proteins, and different estimated parameters

**Fig. 1:**
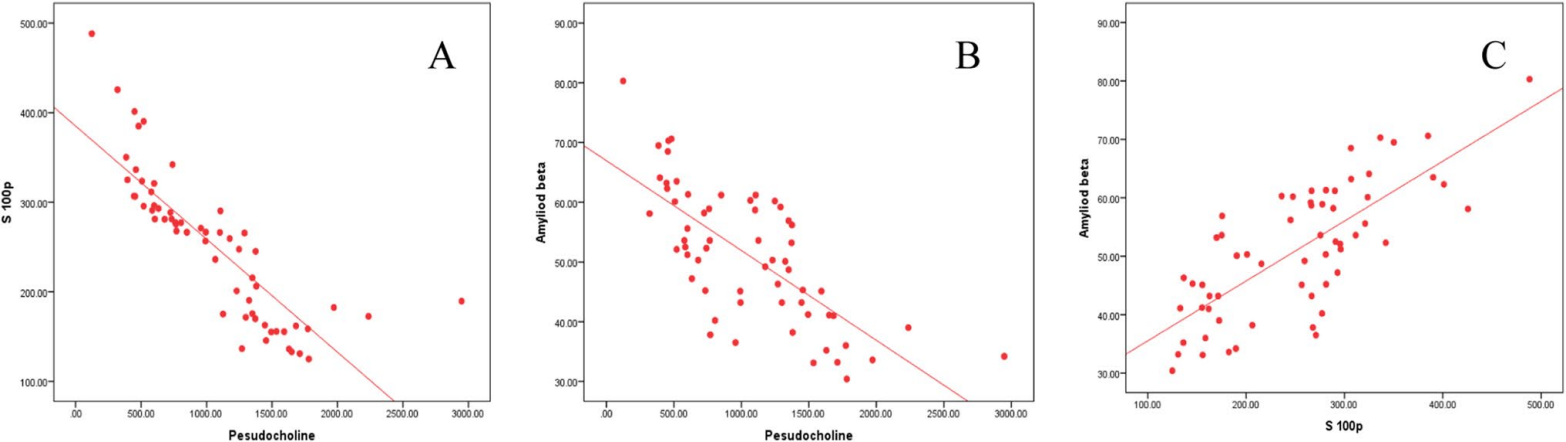
Pearson’s correlation analysis between serum levels of estimated parameters in the organophosphate-intoxicated group

Pearson’s analysis indicated that serum S100B was positively correlated with amyloid β. In contrary the activity of pseudocholinesterase was negatively correlated with both of S100B and amyloid β. With the increase of OP severity, serum S100B and amyloid β levels progressively raised; in contrast, the pseudocholinesterase activity was gradually reduced (Table 6 and Fig. 1 A, B, C).

**Table 6:** Correlation between targeted parameters in different groups

	Moderate group				Severe group			
	S100B		Aβ		S100B		Aβ	
	*r*	*p*	*r*	*p*	*r*	*p*	*r*	*p*
S100B	-	-	0.690	0.000	-	-	0.683	0.000
Amyloid β	0.690	0.000	-	-	0.683	0.000	-	-
ALT	0.126	0.508	0.138	0.466	0.115	0.545	0.105	0.582
AST	− 0.427	0.019	− 0.175	0.354	0.084	0.658	0.156	0.410
Urea	− 0.126	0.508	− 0.436	0.016	0.128	0.499	− 0.033	0.582
Creatinine	− 0.210	0.265	− 0.161	0.395	− 0.148	0.435	− 0.241	0.199
Acetylcholine	0.417	0.022	0.656	0.000	0.738	0.000	0.721	0.000
Malondialdehyde	0.457	0.011	0.404	0.027	0.657	0.000	0.779	0.000
PCE	− 0.626	0.000	− 0.858	0.000	− 0.875	0.000	− 0.762	0.000

## Discussion

The management of OP intoxication in developing nations is not efficient. As a result, it has been envisioned that up to 25 million agricultural workers globally unintentionally poison themselves with pesticides each year (Alavanja 2009).

Because pesticides are widely and readily available, they are frequently used for self-harm, i.e., causes of suicide in humans due to the liberalization of regulatory agencies in developing countries compared to developed countries, which keep strict monitoring by regulatory agencies (Dubey et al. 2016).

Gender-wise distribution of studied population was predominantly male (60%) compared to female (40%). The most dominant age was about 18 years. Additionally, the majority of OP poisoning occurred due to suicidal ingestion (83%). The reasons for suicides were as follows: is influenced by social inequities, peer pressure, pop culture, financial disparity, lack of opportunities, fear of missing out, family problems, educational misadventures, or failures (Amir et al. 2020).

The mortality rate was 5% which was comparable to previous studies done in Egypt (5.5%) (Abdel basser et al. 2021) and Nepal (5.9%) (Koirala et al. 2013). The main cause of death was related to respiratory failure transport from rustic area after ingestion of large amount of OPs leading to improper preliminary resuscitation measures that aggravate the problem. The estimated average time for admission to the PCC is about 6 h which masters that rapid admission was an indispensable factor for the high survival rate of the enrolled subjects where early referrals lead to proper and effective intervention (Mahmoud et al. 2021). Hypoperfusion of the central nervous system due to neuropathy presents the potential for development of respiratory failure leading to varieties of clinical signs and symptoms. Myosis was recorded as the most frequently encountered sign in our study (98%) and diarrhea was the most common symptom (50%). The deviation in our medical presentation among different studies may be related to the implicated OP, the amount absorbed, and the route of exposure.

The Peradeniya OP Poisoning Scale established by Senanayake et al. (1993) for categorization of OP-intoxicated sufferers identifies clinical parameters that reflect the muscarinic, nicotinic, and central effects of OP compounds that could grade severity without laboratory investigations. We rely upon this scale as it does not base on patient cooperation and hence can be applied to all groups of patients without the need of their partnership.

The possible mechanism for the elevation of liver transaminases is unclear, but disturbances in the antioxidant defense system, oxidative stress, apoptosis, and mitochondrial and microsomal metabolism seem to be culprits in the development of hepatoxicity (Senatathne et al. 2022).

Renal damage following OP exposure was considered a rare phenomenon with the development of clinical symptoms of poisoning (Anormallikleri,20,210). The nephrotoxic effects after OP poisoning in humans had been published (Risal et al. 2019;Wedin 1992). More attention has been challenged to the nephrotoxicity of organophosphate toxicants.

Diverse research cautioned that both renal circulation and electrolyte excretion had been under partial cholinergic manipulation so that partial exposure to cholinesterase can also disrupt the renal features. Also, it has been proven that OP poisoning regularly led to pathophysiological renal damage. The present study revealed that perspicuous increase in the level of urea and creatinine in different groups of OP-intoxicated patients may be due to degeneration in the tubule epithelial cells and epithelial cell loss and atrophy in the glomerular structures (Kaya et al. 2018).

Oxidative stress is considered one of the possible mechanisms of OP toxicity. It has become a focus of toxicological research because it is considered a critical pathophysiological mechanism in different human pathologies (Lukaszewicz 2010). The present study revealed a significant increase in MDA level, the biomarker of oxidative stress in the OP-exposed group in comparison to controls. The increased MDA level may lead to peroxidative damage deteriorating the structural and functional integrity of neuronal membrane. It inhibits cholinesterase activity initiating cellular dysfunction.

The changes in the levels of estimated routine parameters were dependent on severity score measured by a scale and the activity of cholinesterase that demonstrates the potential of analyzing liver and kidney candidates as a marker of severity.

Clinical suspicion of OP poisoning should be raised if there is no history of exposure or ingestion. Some OP has a distinct garlic or petroleum odor that may aid in diagnosis. Confirmation of organophosphate poisoning is based on the measurement of cholinesterase activity. But cholinesterase activity does not always correlate with severity of clinical illness. Moreover, a variety of conditions can result in falsely lowered cholinesterase activity (Eddleston et al. 2008). Neurochemical studies have confirmed that some specific proteins, S100B protein and Aβ protein, can be used as markers in the diagnosis of some neurological diseases. Various clinical investigations have demonstrated the feasibility of using these protein markers for evaluating the pathological changes in the nervous system (Ramaker 2016). In this study, serum S100B was used to detect their value as an early marker of severity of OP exposure (Yardan et al. 2013). The role of S100B is a predictor of neurologic complications in patients with organophosphate poisoning; serum S100B was higher in patients than in the control group. Pseudocholinesterase was reduced in the moderate and severe group pseudocholinesterase level. These data suggested that S100B may be a useful marker in the assessment of clinical severity and prediction of mortality in acute OP (Oreby and El madah 2017). The Aβ protein is an acute phase protein; its elevation in serum is one of the most rapid and intense sensitive markers in neurodegenerative disease (Jenna et al 2019). It was found that Aβ protein showed a significant elevation in intoxicated groups versus the control one. This is in agreement with Sarkar et al. (2017) and Iddi et al. (2019). In the current study, S100B showed to be more sensitive and specific and has better specificity, PPN, NPV, diagnostic accuracy, and AUC than Aβ protein for severity of OP patients; the measure of both proteins could add important information to clinical judgment in establishing a final diagnosis of OP patients. Concerning the correlations between all parameters in patient groups, it was obtained that there were strong correlations between all markers of nerve cell degeneration (S100B, Aβ protein, ACh, and PChE) and all markers of muscle cell degeneration and liver enzymes. It was concluded that the changes in the levels of S100B and Aβ can be used as rapid sensitive and specific markers for OP intoxication severity for fast management.

## Data Availability

All datasets generated or analyzed during this study are included in the manuscript.

## Abbreviations

Ops: Organophosphates
Aβ: Amyloid β
MDA: Malondialdehyde
AUC: Area under curve
CNS: Central nervous system
GIT: Gastrointestinal tract
ALT: Alanine transaminase
AST: Aspartate transaminase
ACh: Acetylcholine
PChE: Pseudocholinesterase

## References

[CR1] Abdel Gad Abdel Raheem A EFYF (2021). Clinical profile and outcome of acute organophosphate poisoning in children of upper Egypt: a cross-sectional study. BMC Pediatr.

[CR2] Alavanja MC (2009). Introduction: pesticides use and exposure, extensive worldwide. Rev. Environ Health.

[CR3] Amir A, Raza A, Qureshi T, Mahesar GB, Jafferi S, Haleem F, Ali KM (2020). Organophosphate poisoning: demographics, severity scores and outcomes from national poisoning control centre. Karachi Cureus.

[CR4] Anand S, Singh S, Nahar SU, Bhalla A, Paul SY, Singh D (2009). Cardiac abnormalities in acute organophosphate poisoning. ClinToxicol (phila).

[CR5] Banerjee D, Singh S (2019)Organophosphrous pesticide exposure and Alzheimer disease.acta scientific medical sciences.3(1):1–2

[CR6] Braak H, Thal DR, Ghebremedhin E, Del Tredici K (2011). Stages of the pathologic process in Alzheimer disease: age categories from 1 to 100 years. J NeuropatholExpNeurol.

[CR7] Ceja GHR, Torres ED, Orres JH, Ornelas AV, Salazar FJ 2020 Effect of structure and function of paraoxonase (poN-1) on organophosphate pesticide metabolism.Biocell 44(3):363–360

[CR8] Chen J, Yan C, Zhang X, Wang F, Chuai X (2022) Clinical value of serum neuron-specific enolase combined with serum S100B protein in the diagnosis of systemic lupus erythematosus. Contrast media & molecular imaging 9390991 10.1155/2022/9390991 PMC9110203 35615727

[CR9] Dubey T, Yadav S, Kawre KK (2016). Correlation of severity of OP poisoning as assessed by Peradeniya OP poisoning scale with serum amylase and CPK level.. Int. J. Contemp Med Res.

[CR10] Eddleston M, Buckley NA, Eyer P, Dawson AH (2008) Management of acute OP pesticide poisoning. Lancet 317:597-607 10.1016/S0140-6736(07)61202-1 PMC2493390 17706760

[CR11] Henry R, Cannon D,Winkelman J (1960) Clinical chemistry-principles and technics 2nd ed Harper and Row, Hagerstown, MD 543-552

[CR12] Iddi S, Li D, Aisen PS, Rafii MS, Thompson WK, Donohue MC (2019).Predicting the course of Alzheimer’s progression. Brain informatics (1):6–28 10.1186/s40708-019-0099-0 PMC6598897 31254120

[CR13] Jacob K (2017). Suicide prevention in low-and middle-income countries: part perceptions, partial solutions. Br J Psychiatr.

[CR14] Ramaker J M,Robert SC,Tracy LS,Hanil Q, Philip FC(2019) Amyloid precursor proteins are dynamically trafficked and processed during neuronal development. Mol Neurosc 10:338–339 10.3389/fnmol.2016.00130 PMC5122739 27932950

[CR15] Karki P, Ansari JA, Bhandary S, Koirala S (2004). Cardiac and electrocardiographical manifestations of acute organophosphate poisoning. Singapore Med J.

[CR16] Kassirer J, Brand D, Schwartz W (1971). An automated system for data processing in the metabolic balance laboratory. Comput Biomed Res.

[CR17] Kaya Y, Bas O, Hanci H, Cankaya S, Nalbant I, Odaci E, AvniUydu H, Aslan A (2018). Acute renal involvement in organophosphate poisoning: histological and immunochemical investigations. Ren Fail.

[CR18] Koirala DP, Rao KS, Malla KK, Malla T (2013). A study of clinical features, management and outcome of organophosphate and carbamate poisoning in children. J Nepal Paediatr Soc.

[CR19] Lukaszewicz-Hussain A (2010). Role of oxidative stress in organophosphate insecticide toxicity–short review. *Pestic*. Biochem Physiol.

[CR20] Mahmoud E,abdel Salam M,Halawa H, Hafez (2021) Red cell distribution width as predictor of severity and out come of acute organophosphorus poisoned cases admitted to poison control center ain shams University Hospital (a prospective study) .Ain shams J Forensic Med Clin Toxicol 37(2):26_33

[CR21] Manoranjitham SD, Rajkumar AP, Thangadurai P, Prasad J, Jayakaran RS (2010). Risk factors for suicide in rural south India. Br J Pyschiatr.

[CR22] Miller-Thomas MM,Slipe Al,Benzinger TL,Mcconathy J,Connolly Schwetye KE (2016) Multimodality review of amyloid-related diseases of the central nervous system. Radiographics : a review publication of the Radiological Society of North America 36(4): 1147–63 10.1148/rg.2016150172 PMC4976469 27399239

[CR23] Noshad H, Ansarin K, Ardalan MR, Ghaffari AR, Safa J Nezami N (2007) Respiratory failure in organophosphate insecticide poisoning. Saudi Med J 28:405‑7 17334469

[CR24] Oreby M, El-Madah E (2017). Prediction of acute organophosphate poisoning using Glasgow Coma Sale, serum cholinesterase and S100B. Ain Shams J Foren Med Clin Toxicol.

[CR25] Ramaker JM, Cargill RS, Swanson TL, Quirindongo H, Cassar M, Kretzschmar D, Copenhaver PF (2016). Amyloid precursor proteins are dynamically trafficked and processed during neuronal development. Front Mol Neurosci.

[CR26] Risal P, Lama S, Thapa S, Bhatta R, Karki RK (2019). Cholinesterase and liver enzymes in patients with organophosphate poisoning. J Nobel Med Coll.

[CR27] Sarkar B, Dhiman M, Mittal S, Mantha AK (2017). Curcumin revitalizes amyloid β (25–35)-induced and organophosphate pesticides pestered neurotoxicity in SH-SY5Y and IMR-32 cells via activation of APE1 and Nrf2. Metab Brain Dis.

[CR28] Senanayake N, de Silva HJ, Karalliedde LA (1993). Scale to assess severity in OP intoxication: POP scale. Hum Exp Toxicol.

[CR29] Senarathna L, Jayamanna SF, Kelly PJ, Buckley NA, Dibley MJ, Dawson AH (2012) Changing epidemiologic patterns of deliberate self poisoning in a rural district of Sri Lanka. BMC Public Health 12–593 10.1186/1471-2458-12-593 PMC3458971 22852867

[CR30] Senarathne R, Hettiaratchi U, Athiththan L, Peiris H, Sarathchandra C, Senanayake H, Weerawansa P, Siribaddana S (2022). Selected liver markers in predicting the severity of organophosphate and carbamate poisoning. J Environ Public Health.

[CR31] Tareg AB (2001)Organophosphate and carbamate insecticides. Clinical environmental health and toxic exposures and GR Krieger. Philadelphia, Lippincott Williams & Wilkins 1046–57

[CR32] Tokshilykova AB, Sarkulova ZN, Kabdrakhmanova GB, Utepkaliyeva AP, Tleuova AS Satenov ZK (2020) Neuron-specific markers and their correlation with neurological scales in patients with acute neuropathology. J Mol Neurosci 70(8):1267–1273 10.1007/s12031-020-01536-5 32350763

[CR33] Wedin GP (1992) Nephrotoxicity of anticholinesterases. In Clinical and experimental toxicology of OP and carbomates ballantyeye B mars TC.Eds 195–202

[CR34] Yardan T, Baydin A, Acar E, Ulger F, Aygun D, Duzgun A, Nar R (2013). The role of serum cholinesterase activity and S100B protein in the evaluation of organophosphate poisoning. Hum Exp Toxicol.

